# Assessing the Impact of Maternal Drinking During and After Pregnancy

**Published:** 1997

**Authors:** Sandra W. Jacobson

**Affiliations:** Sandra W. Jacobson, Ph.D., is a research professor in the Department of Psychology, Wayne State University, and in the Department of Obstetrics and Gynecology, Wayne State University School of Medicine, Detroit, Michigan

Recent findings from longitudinal followup studies of adolescents and adults with fetal alcohol syndrome (FAS) indicate that the deficits associated with this disorder are long lasting and pervasive. In addition, followup data from several large, prospective studies of cohorts representing a broad range of alcohol exposure levels have confirmed that although FAS represents the severe end of a continuum of birth defects, moderate^1^ levels of alcohol intake produce physical and neurobehavioral deficits that are similar to, but less severe than, FAS (e.g., Streissguth et al. 1986, 1994; Coles et al. 1991; Jacobson and Jacobson 1994; Goldschmidt et al. 1996).

Accumulating evidence shows that many FAS deficits can be detected at infancy and remain through adolescence into adulthood. In particular, an abnormally small head circumference (i.e., microcephaly) and intellectual problems persist as an affected child matures, whereas behavioral, emotional, and social problems can become more pronounced (e.g., Streissguth et al. 1991). In addition, the long-term clinical consequences in terms of psychopathology and social maladjustment are only recently being recognized and persist even in people with FAS who were raised in a stable, supportive environment (Steinhausen et al. 1993). Incidences of maladaptive behaviors, such as poor judgment, failure to consider the consequences of one’s actions, and difficulty perceiving social cues, are common, as are legal problems resulting from sexual misconduct, drunk driving, shoplifting, and other socially inappropriate behaviors (LaDue et al. 1992).

In a recent long-term followup study of a Seattle cohort of adolescents and adults with FAS, Streissguth and colleagues (1996) distinguished between prenatal, or “primary,” disabilities, which reflect central nervous system (CNS) dysfunctions inherent in the FAS diagnosis, and “secondary” disabilities, with which a person is not born and which presumably could be ameliorated through intervention. Examples of secondary disabilities include mental health problems, disrupted schooling (e.g., dropping out or being suspended or expelled), trouble with the law (e.g., being charged with or convicted of a crime), inappropriate sexual behavior (e.g., promiscuity), alcohol or drug problems, dependent living as an adult (e.g., an inability to manage money), and problems with employment (e.g., trouble holding a job). Streissguth and colleagues concluded that legal problems and other secondary disabilities occur frequently in adolescents and adults with FAS, with incidences ranging from approximately 30 to 94 percent for various types of secondary disabilities among the study sample.

## Prenatal Versus Postnatal Effects

This article aims to demonstrate the need to differentiate the impact of prenatal drinking from the impact of the environment in which the child is raised when assessing neurobehavioral and other outcomes in children whose mothers drank both during and after pregnancy. In the 20 years since FAS was first identified, a major body of research has been compiled on the effects of prenatal alcohol exposure as well as the impact of being raised by an alcoholic^2^ parent. Distinguishing between the effects of prenatal exposure and the effects of postnatal environment often presents a major methodological challenge to researchers, however, because women frequently drink both during and after pregnancy (i.e., prenatal and postnatal drinking are moderately related).

**Figure 1:**
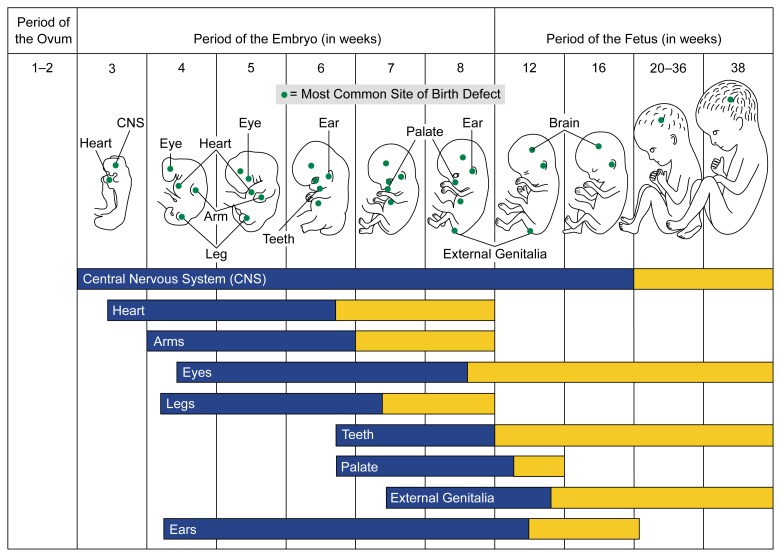
Vulnerability of the fetus to defects during different periods of development. The black portion of the bars represents the most sensitive periods of development, during which alcohol-induced (i.e., teratogenic) effects on the sites listed would result in major structural abnormalities in the child. The gray portion of the bars represents periods of development during which physiological defects and minor structural abnormalities would occur. SOURCE: Adapted from Moore and Persaud 1993.

An association between drinking during pregnancy and neurobehavioral function in infants and children normally is interpreted as teratogenic (i.e., attributable to a direct effect of alcohol exposure on fetal CNS development). The specific body systems affected by alcohol exposure and the resultant outcomes depend on when exposure occurs during the prolonged period of CNS sensitivity to alcohol (see figure 1). For example, first-trimester exposure is related to craniofacial anomalies (e.g., Coles 1994), whereas the effects on growth—particularly postnatal growth—are related to alcohol exposure later in pregnancy (e.g., Coles et al. 1991; Day et al. 1991; Jacobson, J.L., et al. 1994). Experimental studies with laboratory animals have demonstrated the role of timing of bingelike alcohol exposure in inducing specific structural and behavioral deficits (e.g., Goodlett and Stevenson 1994; West and Goodlett 1990). However, little is known about the timing of exposure for many important neurobehavioral effects in humans, such as deficits in attention span or information-processing speed.

One alternative explanation—that an observed deficit is attributable to the socioenvironmental consequences of being raised by a drinking mother—can be evaluated by examining the relationship of the deficit to postnatal maternal alcohol use. In cases where the mother drinks both during and after pregnancy, however, it may not always be possible to determine the degree to which observed deficits are attributable to teratogenic versus socioenvironmental factors. Statistical analyses that include variables related to both prenatal and postnatal drinking behavior may sometimes obscure true prenatal effects and result in the failure to recognize a true effect or in an understatement of the magnitude of the effect (i.e., type II error).

## Two Approaches to Assessment

**Figure 2:**
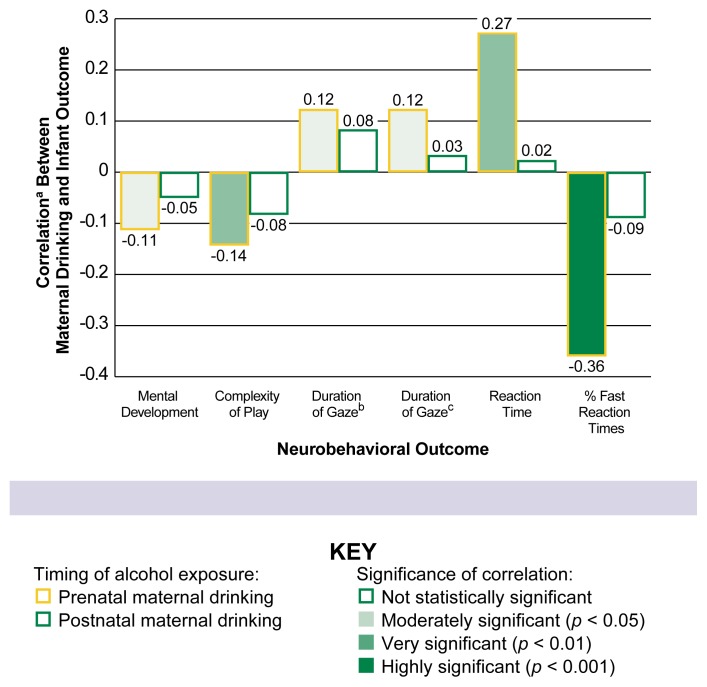
Effects of alcohol exposure on infant outcomes by prenatal and postnatal drinking. Infants whose mothers drank during or after pregnancy were assessed on a variety of neurobehavioral tests. After adjusting for the influence of variables that potentially could cause or prevent the infant outcomes (i.e., confounding variables), the results indicated that the detrimental effects of drinking during pregnancy were statistically significant for all of the outcomes assessed. (The intensity of the bar shading indicates the significance level—i.e., the darker the bar, the stronger the relationship between maternal drinking and infant outcome.) In contrast, none of the infant outcomes was significantly related to postnatal maternal drinking. ^a^Correlation measures the degree to which maternal drinking is related to infant outcomes. The maximum positive correlation is +1.00, and the maximum negative correlation is −1.00. Results close to these extremes indicate a high degree of predictability, whereas results closer to zero indicate little relationship between the two variables. ^b^As measured by the Fagan Test of Infant Intelligence. ^c^As measured by a cross-modal transfer test.

Two analytical approaches have been used to assess the impact of prenatal versus postnatal drinking on the child. One approach was used in a prospective longitudinal study (Jacobson, J.L., et al. 1993; Jacobson, S.W., et al. 1993) conducted in Detroit, Michigan, that investigated the effects of prenatal and postnatal alcohol exposure on infant neurobehavioral outcomes. Infants were assessed on the Bayley Scales of Infant Development (Bayley 1969), a complexity of play measure (Belsky et al. 1984), and three infant information-processing tests:

(1) the Fagan Test of Infant Intelligence (Fagan and Singer 1983), (2) a test of cross-modal transfer (Rose and Wallace 1985), and (3) the Visual Expectancy Paradigm (Haith et al. 1988). Infants with moderate prenatal alcohol exposure performed more poorly than less-exposed infants on most of these tests, even after controlling for potential confounding variables (see figure 2). For example, prenatally exposed infants received lower scores on the mental development scale of the Bayley Scales of Infant Development (Jacobson, J.L., et al. 1993) and on a test of play complexity (Jacobson, S.W., et al. 1993). Prenatal alcohol exposure also was associated with slower response times on the Visual Expectancy Paradigm, which directly assessed the infants’ reaction time as they shifted their gaze back and forth at an image flashed on a screen (Jacobson, S.W., et al. 1994). This result suggests slower information processing in prenatally exposed infants. Similarly, these infants demonstrated slower processing speed measured in terms of the length of their gaze (i.e., visual fixation) as they studied an object or picture on both the Fagan Test of Infant Intelligence and the cross-modal transfer test. Short looks, which are associated with more rapid information processing, have been found to predict a higher childhood IQ (Colombo 1993).

As seen in figure 2, none of the neurobehavioral deficits detected during infancy was significantly related to postpartum drinking by the mother or caregiver, suggesting that these deficits were related specifically to prenatal alcohol exposure. Because postpartum drinking levels were unrelated to infant outcomes, they could not be potential confounding variables.^3^ Therefore, postpartum drinking levels were not included in analyses assessing the impact of prenatal exposure on outcome, even though mothers who drank during pregnancy were likely to drink afterward as well (i.e., prenatal and postnatal drinking were moderately correlated^4^).

A second analytical approach for assessing the effects of drinking during and after pregnancy was used in a longitudinal study conducted in Atlanta, Georgia (Coles et al. 1991). The deficits in intellectual functioning seen in children heavily exposed to alcohol throughout pregnancy continued to be evident even after the analyses statistically controlled for current drinking reported by the mothers or caretakers. Children exposed throughout pregnancy also were more often described as showing higher levels of negative externalizing behaviors, including destructive, inattentive, aggressive, and nervous or overactive behaviors; inappropriate social behavior; and poor social competence. These deficits likewise persisted after current caregiver drinking was controlled (Brown et al. 1991). In contrast, the impact of prenatal alcohol exposure on the child’s internalizing behavior (specifically, depression) was no longer significant when the caretaker’s current drinking was controlled. Thus, the child’s depression was attributed at least in part to problems in the postnatal environment.

A similar pattern of results was seen when Brown and colleagues (1991) examined sustained attention. They noted that a formerly significant deterioration of attention span detected in the children of mothers who drank heavily throughout pregnancy was no longer significant when current alcohol use was held constant. Thus, the researchers concluded that this effect derived from the consequences of the current caretaking environment. Alternatively, however, the prenatal and postnatal alcohol exposure measures in these instances could have been too confounded to determine which was the true predictor of the outcomes. The effects on attention cannot be conclusively attributed to the current caretaking environment, unless the impact of current drinking persists after controlling statistically for the influence of the prenatal exposure.

The data from these studies are consistent with findings demonstrating that cognitive performance is less affected by alcohol exposure in infants and children whose mothers stop drinking in early pregnancy, despite the mothers’ resumption of alcohol use after giving birth (e.g., Rosett et al. 1980). Thus, these studies show that although some secondary psychopathology or deficits are attributable to being raised by a mother whose alcohol abuse problems may prevent her from providing an optimal and stable home environment, several specific cognitive and behavioral deficits linked to prenatal alcohol exposure appear to reflect CNS damage.

## Conclusions

The impact of being raised by an alcoholic parent has been examined extensively in the research on children of alcoholics (COA’s), but few studies have compared the effects of being raised by an alcoholic father versus an alcoholic mother. Most COA research has focused on children whose fathers have problems with alcohol abuse or alcoholism, but whose mothers do not, in order to exclude the effects of alcohol exposure attributable to maternal drinking during pregnancy. Little is known about the impact of these nonalcoholic mothers’ drinking habits on their children. As previously noted, however, recent findings (Jacobson and Jacobson 1994) have detected prenatal alcohol effects at moderate levels of alcohol consumption (i.e., between 3.5 and 7.0 ounces of absolute alcohol or the equivalent of 7 to 14 standard drinks per week) by pregnant women not considered to have a serious drinking problem. Thus, even though a mother is not an alcoholic, her child may not be spared the effects of prenatal alcohol exposure. Most likely, however, the pattern of neurobehavioral deficits will differ when such deficits result from direct fetal exposure rather than when they are paternally transmitted or postnatally incurred.

The studies cited in this article illustrate the need to distinguish the effects of drinking during pregnancy from the consequences of being raised by a drinking parent. The methods described here reflect some of the advances in this research area that have enabled investigators to better distinguish between the harmful effects of prenatal alcohol exposure and the additional impairment that may be incurred in an environment in which one or both parents drink heavily. Current research is beginning to explore complex models and is attempting to identify specific factors (i.e., moderators) that may buffer or increase the magnitude of the damage incurred by alcohol exposure.

## Footnotes

1 A “moderate” level of alcohol intake usually refers to an average daily consumption of 0.5 to 0.99 ounce of absolute alcohol or the equivalent of 1 to 1.99 standard drinks.

2 The terms “alcoholism” and “alcoholic” as used in this article are summary terms for the diagnoses of alcohol abuse and alcohol dependence. The term “alcohol abuse” refers to abnormal patterns of drinking that result in detrimental effects on health, social problems, or both.

3 Confounding variables are those variables that can cause or prevent the outcome of interest (in this case, neurobehavioral deficits). Adjustments must be made for confounding variables in order to distinguish their effects from those of the variable under investigation (in this case, drinking during pregnancy).

4 Correlation coefficient (*r*) = 0.49; *p* < 0.001.
